# Phenotypic characterization of people at risk of atrial fibrillation: protocol for the FIND-AF longitudinal cohort study

**DOI:** 10.1093/eurjpc/zwae303

**Published:** 2024-09-25

**Authors:** Ali Wahab, Ramesh Nadarajah, Catherine Reynolds, Sheena Bennett, Edisemi Ambakederemo, Mohammad Harris, Tanina Younsi, Tobin Joseph, Keerthenan Raveendra, Adam Smith, Asad Bhatty, Gregory Y H Lip, Peter P Swoboda, Jianhua Wu, Chris P Gale

**Affiliations:** Leeds Institute of Data Analytics, Clarendon Way, University of Leeds, Leeds LS2 9DA, UK; Leeds Institute for Cardiovascular and Metabolic Medicine, University of Leeds, Leeds LS2 9DA, UK; Department of Cardiology, Leeds Teaching Hospitals NHS Trust, Leeds LS1 3EX, UK; Leeds Institute of Data Analytics, Clarendon Way, University of Leeds, Leeds LS2 9DA, UK; Leeds Institute for Cardiovascular and Metabolic Medicine, University of Leeds, Leeds LS2 9DA, UK; Department of Cardiology, Leeds Teaching Hospitals NHS Trust, Leeds LS1 3EX, UK; Leeds Institute of Data Analytics, Clarendon Way, University of Leeds, Leeds LS2 9DA, UK; Leeds Institute for Cardiovascular and Metabolic Medicine, University of Leeds, Leeds LS2 9DA, UK; Leeds Institute for Cardiovascular and Metabolic Medicine, University of Leeds, Leeds LS2 9DA, UK; Leeds Institute for Cardiovascular and Metabolic Medicine, University of Leeds, Leeds LS2 9DA, UK; Leeds Institute of Data Analytics, Clarendon Way, University of Leeds, Leeds LS2 9DA, UK; Leeds Institute for Cardiovascular and Metabolic Medicine, University of Leeds, Leeds LS2 9DA, UK; Department of Cardiology, Leeds Teaching Hospitals NHS Trust, Leeds LS1 3EX, UK; Leeds Institute of Data Analytics, Clarendon Way, University of Leeds, Leeds LS2 9DA, UK; Leeds Institute for Cardiovascular and Metabolic Medicine, University of Leeds, Leeds LS2 9DA, UK; Faculty of Medicine and Health, University of Leeds, Leeds LS2 9JT, UK; Leeds Institute of Data Analytics, Clarendon Way, University of Leeds, Leeds LS2 9DA, UK; Leeds Institute for Cardiovascular and Metabolic Medicine, University of Leeds, Leeds LS2 9DA, UK; Leeds Institute of Data Analytics, Clarendon Way, University of Leeds, Leeds LS2 9DA, UK; Leeds Institute for Cardiovascular and Metabolic Medicine, University of Leeds, Leeds LS2 9DA, UK; Liverpool Centre for Cardiovascular Science at University of Liverpool, Liverpool John Moores University, Liverpool Heart and Chest Hospital, Liverpool, UK; Danish Center for Health Services Research, Department of Clinical Medicine, Aalborg University, Aalborg, Denmark; Leeds Institute for Cardiovascular and Metabolic Medicine, University of Leeds, Leeds LS2 9DA, UK; Faculty of Medicine and Health, University of Leeds, Leeds LS2 9JT, UK; Department of Cardiology, Mid Yorkshire Teaching NHS Trust, Aberford Road, Wakefiled WF1 4DG, UK; Wolfson Institute of Population Health, Queen Mary University of London, Charterhouse Square, London EC1M 6BQ, UK; Leeds Institute of Data Analytics, Clarendon Way, University of Leeds, Leeds LS2 9DA, UK; Leeds Institute for Cardiovascular and Metabolic Medicine, University of Leeds, Leeds LS2 9DA, UK; Department of Cardiology, Leeds Teaching Hospitals NHS Trust, Leeds LS1 3EX, UK

**Keywords:** Protocol, Atrial fibrillation, Prevention, Primary care, Phenotyping, Cohort, Randomized clinical trial, Multimodality imaging

## Abstract

**Aims:**

The Future Innovations in Novel Detection of Atrial Fibrillation (FIND-AF) longitudinal cohort study is a multi-centre prospective cohort study of patients identified at risk of atrial fibrillation (AF). The aim of the FIND-AF longitudinal cohort study is to provide multi-modal phenotypic characterization of these patients.

**Methods and results:**

A total of 1955 participants identified as at risk of AF by the FIND-AF algorithm from primary care electronic health record (EHR) data, aged 30 years and above and eligible for oral anticoagulation, will be recruited between October 2023 and November 2024 to receive home-based intermittent electrocardiogram monitoring. About 500 participants without diagnosed AF will then undergo cross-sectional phenotypic characterization including physical examination, symptoms assessment, serum blood biomarkers and echocardiography, and non-stress cardiac magnetic resonance imaging. Longitudinal information about cardio–renal–metabolic–pulmonary outcomes will be ascertained from linkages to EHR data. The study is funded by the British Heart Foundation (CC/22/250026). The study has ethical approval (North West—Greater Manchester South Research Ethics Committee reference 23/NW/0180). Findings will be announced at relevant conferences and published in peer-reviewed journals in line with the funder’s open-access policy.

**Conclusion:**

The FIND-AF multi-centre prospective longitudinal cohort study aims to (i) provide evidence for the impact of comorbidities on AF genesis, (ii) uncover actionable targets to prevent AF, and (iii) act as a platform for cohort randomized clinical trials that investigate enhanced detection and prevention of AF.

## Graphical Abstract

**Graphical Abstract:**
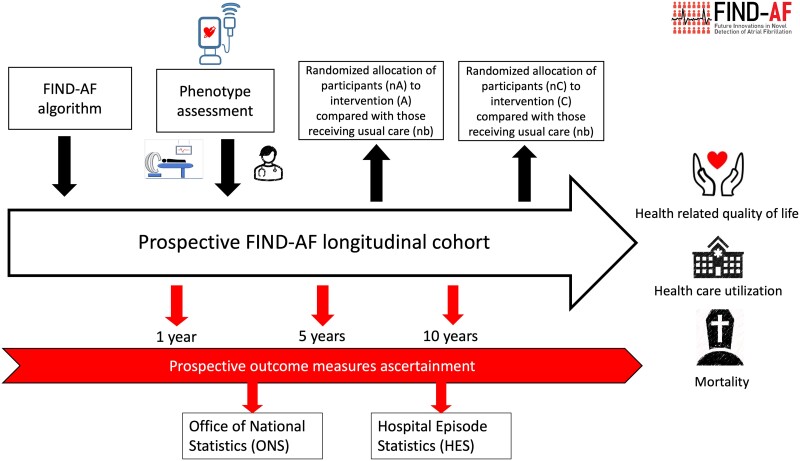

## Introduction

Atrial fibrillation (AF) is a major global public health issue. It is the most common sustained cardiac arrhythmia worldwide,^1^ and incident cases per year outstrip the four most common causes of cancer combined in the UK.^2^ Atrial fibrillation confers an increased risk of stroke, heart failure, cognitive decline, and death and is associated with quantifiable impairment in quality of life.^1^ Once diagnosed, most patients will require lifelong treatment and rate or rhythm control for symptom relief and oral anticoagulation to mitigate the elevated risk of stroke. Within 1 year of diagnosis, one-fifth of patients have died, and the rate of unscheduled hospitalization is increasing by 5% per annum.^2^ It is estimated that AF accounts for 1.6% of the current UK National Health Service (NHS) expenditure and may account for up to 4.3% by 2040.^3^

To tackle the growing burden of AF and its consequences, health services will need to prioritize treatment upstream towards the early detection and prevention of AF.^4^ Yet, previous efforts to detect AF early, or prevent the manifestation of the arrhythmia, have been limited by an inability to accurately identify a higher risk cohort. Recently, the Future Innovations in Novel Detection of Atrial Fibrillation (FIND-AF) algorithm has been demonstrated to accurately predict short- and long-term absolute risk of AF^5^ and has been implemented through routine primary care records in the UK.

There is limited information about the clinical, biochemical, and imaging traits of individuals at risk of AF—‘pre-AF’—and their short- and long-term outcomes. Moreover, the development and characterization of a prospective cohort of patients at high risk of AF offer the opportunity to embed randomized clinical trials (RCTs),^6^ testing interventions to mitigate AF risk and modify health outcomes.

### Study aims

The aims of the FIND-AF longitudinal cohort study are as follows:

- to perform multi-modal cross-sectional characterization of participants at risk of AF (those with higher predicted risk of AF, but without a diagnosis of AF);
- to collect longitudinal health outcomes in these individuals; and
- to provide a unique platform for subsequent research studies including RCTs.

This will enable enhanced understanding of how comorbidity modification influences the individual-level AF occurrence, identification of novel targets to prevent AF, and conduct of interventional studies to prevent or detect AF.

## Methods and analysis

### Setting

The FIND-AF longitudinal cohort is a multi-centre, prospective cohort study that will be conducted in the NHS of England (*Graphical Abstract*). A total of seven NHS primary care general practices in the National Institute for Health and Care Research (NIHR) Research Delivery Network Yorkshire and Humber region have participated in the study, with planned expansion across geographically diverse sites in the UK.

### Populations and consent

Participants were recruited into the FIND-AF study between October 2023 and September 2024 with participants aged ≥30 years, without known AF or atrial flutter, and eligible for oral anticoagulation (men with a CHA_2_DS_2_-VASc score ≥ 2 or women with a CHA_2_DS_2_-VASc score ≥ 3) (NCT05898165).^7^ Individuals receiving any form of anticoagulation and those on the palliative care register are excluded.

Eligible participants were identified by the primary care team via an electronic search of primary care data. The invitation process consists of a text message followed by an information pack in the post including a participant information sheet, consent form, data protection leaflet, and freepost return envelope. All participants were required to provide written informed consent by returning a completed consent form. Consented individuals have had their AF risk estimated using FIND-AF.^7^ All participants have received non-invasive electrocardiogram (ECG) monitoring with a handheld Zenicor-ECG recorder and were asked to record their ECG four times daily (morning, noon, afternoon, and evening) or whenever they have palpitations, for 3 weeks. The study was given ethical approval by the North West—Greater Manchester South Research Ethics Committee (REC reference: 23/NW/0180).

### The FIND-AF longitudinal cohort

Individual FIND-AF risk scores are generated by the application of the prediction model to a patient’s primary care electronic health records (EHRs),^8^ with entry into FIND-AF longitudinal cohort study requiring participants to be at higher risk of AF (FIND-AF scores ≥ 0.00834) and not to have received a diagnosis of AF during the non-invasive ECG monitoring period. Participants with lower FIND-AF risk score (<0.00834) will be excluded from phenotype characterization.

### Baseline and follow-up data

**Figure 1:**
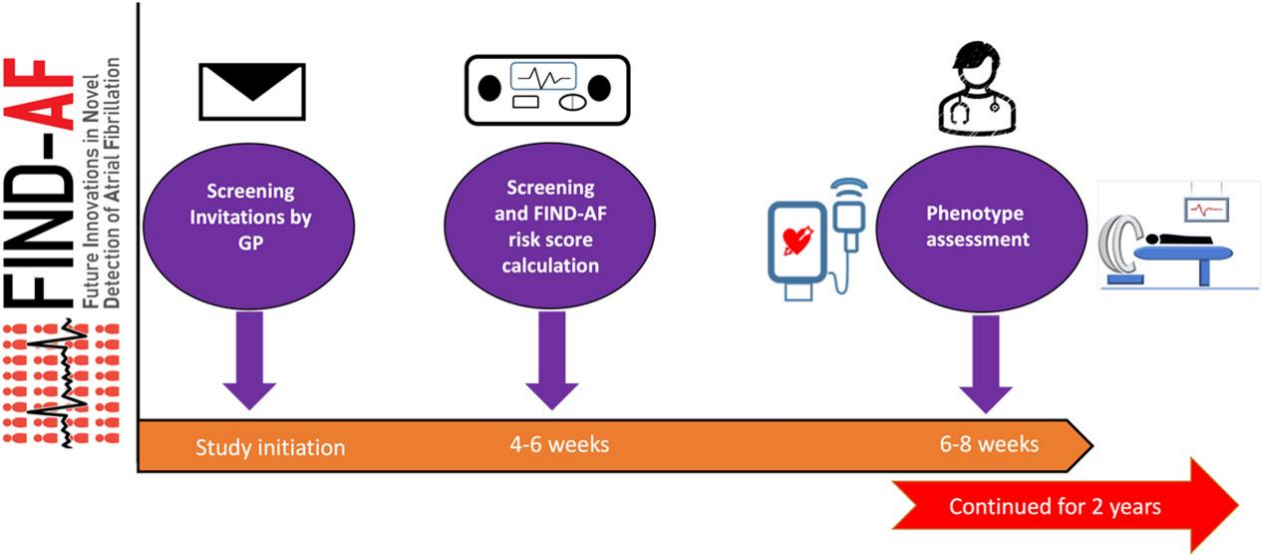
Timeline for phenotypical characterization recruitment.

Individuals at risk of AF are invited for clinical assessment at their primary care centre with the research team for multi-modal phenotyping (*Figure 1*). Currently, 262 participants have been phenotyped, with the aim to assess 500 participants.

Baseline data include each participant’s demographics, observations including height, weight, heart rate, blood pressure, and body mass index, and symptoms. Cardio–renal–metabolic–pulmonary co-morbidities and risk factors (including smoking) will be collected from the participant’s primary care data. Quality of clinical care is evaluated based on the National Institute of Health and Care Excellence (NICE) guidelines for the management of cardiovascular disease, chronic kidney disease, diabetes, and respiratory disease including asthma and chronic obstructive pulmonary disease.^9^ Follow-up for clinical events and life status will be recorded at 6 months, 1 year, 5 years, and 10 years.

### Multi-modal phenotyping

#### Biomarkers

All participants will have their existing routine blood profile reviewed for the preceding 6 months from point of attendance to phenotype assessment. Serum haematology will include full (complete) blood count assessment including haemoglobin, mean cell volume, and platelet count. Biochemistry assessment includes serum urea, creatinine, estimated glomerular filtration rate (eGFR), sodium, potassium, liver function profile, thyroid stimulating hormones, lipid profile, and serum N-terminal prohormone of brain natriuretic peptide levels. Blood analyses will be conducted at local NHS hospital laboratories using standard protocols for analysis and reporting.

#### Echocardiography

Participants will undergo transthoracic echocardiography as part of their phenotype evaluation. This will be undertaken by a British Society of Echocardiography transthoracic echocardiogram accredited cardiology research fellow. Images will be obtained using KOSMOS EchoNous Ultraportable Ultrasound. The left atrium size will be assessed by volumetric analysis indexed to body surface area. The left ventricular internal diameter will be measured in end-diastole and end-systole, respectively. Ventricular systolic function will be estimated visually and by Simpson biplane using in-built KOSMOS Ejection Fraction Artificial Intelligence software. If there are limited views from apical windows that prevent the use of KOSMOS AI, visual estimation will be made for left ventricular ejection fraction assessment. All chamber dimensions will be measured in centimetres and indexed to body surface area. Tissue Doppler imaging will be used to assess diastolic function and right ventricular systolic function. Right ventricular longitudinal and radial function will be assessed through measurement of tricuspid annular plane systolic excursion. All valves will have colour Doppler and continuous and pulse wave Doppler measurements taken from multiple windows. Any new clinically significant features identified from EchoNous will be communicated to the participant’s primary care team. Participants will be notified in the phenotype clinic appointment with further evaluation to be organized by their primary care team.

#### Cardiovascular magnetic resonance

All cardiac magnetic resonance imaging will be undertaken on a 3T system (Siemens Magnetom Prisma, Erlangen, Germany). Participants will be advised to avoid caffeine 24 h prior to their scan. Participants with eGFR < 30 mL/min/1.73cm^2^ will be excluded from cardiac magnetic resonance imaging due to the risk of nephrogenic systemic fibrosis. Participants with previous experience of claustrophobia will also be excluded. The protocol consists of cine imaging, native and post-contrast T1 mapping using a modified Look-Locker sequence, motion-corrected automated in-line perfusion mapping, and motion-corrected bright blood late gadolinium enhancement. An intravenous bolus of 0.05 mmol/kg gadobutrol (Gadovist, Leverkusen, Germany) will be administered 5 mL/s followed by a 20 mL saline flush using an automated injection pump (Medrad MRXperion Injection System, Bayer).

Late gadolinium enhancement will be reported if enhancement is identified in two orthogonal planes or both on dark and bright blood late gadolinium enhancement images. Ischaemic late gadolinium enhancement will be defined if there is sub-endocardial enhancement in typical coronary artery territories. T1 and perfusion maps will be analysed using cvi42 software (Circle Cardiovascular Imaging, Calgary, Canada). Volumetric data including left and right ventricular end-diastolic and end-systolic volumes will be recorded after indexing to body surface area, in addition with other parameters such as left ventricular mass and atrial chamber volumes. All cardiac magnetic resonance images will be independently reviewed by imaging cardiology consultant with the generation of an imaging report for any clinically significant cardiac or extra-cardiac features identified on the research scan. Participants will be made aware of positive features directly by the research team and their parent primary care team notified through the generation of an imaging report with the conclusion of clinically significant findings and further appropriate management suggested.

In addition to the baseline cardiac magnetic resonance imaging protocol, participants will have quantification of left atrial fibrosis using ADAS-3D left atrial image post-processing software (Galgo Medical, Barcelona, Spain). The 3D left atrial images will undergo quality control by two experienced readers prior to post-processing with images deemed poor quality to be excluded from final analysis. The left atrium including pulmonary vein insertion points will be segmented manually in multiple axial planes by drawing mid-atrial wall contours on 3D late gadolinium enhancement images. The artificial intelligence interface will interpolate the intermediate slices for purposes of 3D left atrium reconstructed image excluding left atrium appendage and pulmonary veins beyond ostial levels. The mitral valve annulus will be used to separate the left ventricle cavity from the left atrium, and any enhancement due to its structure will be excluded from the final left atrial fibrosis calculation.

#### Data capture and storage

Participating research team members will input data into an online electronic case report form specific to the cohort, accessible through the investigator's unique username and password provided by the University of Leeds. The collected patient data remain anonymous, and participants are identified with a unique code. Anonymized data are securely stored in a central database, with limited access protected by individual encrypted and coded passwords. Patient-identifying data necessary for follow-up visits are stored separately from data collection. Data storage and sharing abide by the University of Leeds data protection and sharing policies. If and when appropriate, data will be stored in the Leeds Analytics Secure Environment for Research (LASER) within the University of Leeds. The LASER is a Leeds Institute for Data Analytics purpose-built cloud-based platform for hosting sensitive data compliant with ISO 27001 standards and the NHS data security and protection toolkit. Pseudonymized data will be made accessible for analysis with approval from the local data controller (C.P.G.) in a dedicated secure LASER Virtual Research Environment. All individuals with access to these data are required to undergo the University of Leeds information security training and to sign an information security policy before accessing the data. The University of Leeds Information Security Policy is implemented and drawn up in line with ISO 27001.

#### Data quality

The FIND-AF electronic case report form will be cross-checked automatically and manually to ensure data completeness and validity. The University of Leeds will support regulatory and data entry activity at each participating centre, including participant enrolment, complete data entry for baseline, and follow-up assessment, and will also provide continuous support, training, and tracking of progress.

#### Endpoints and linkage to other data

Adherence to NICE guidelines^10^ in primary care during the study period will be investigated. Follow-up for incident cardio–renal–metabolic–pulmonary disease diagnosis, clinical events and life status will be recorded at 6 months, 1 year, 5 years, and 10 years from primary care data, and participants have given consent for their data to be linked to other registries, Hospital Episodes Statistics Admitted Patient Care data and Office for National Statistics (ONS) mortality data. Hospital Episode Statistics is a data warehouse containing details of all Admitted Patient Care, Outpatient Attendances, and Urgent and Emergency Care Activities in England.^11^ It is an administrative database that provides data for the purpose of healthcare analysis to the NHS, government, NICE, and researchers. The Hospital Episode Statistics data are created from data submitted to NHS England as part of the Commissioning Data Sets, which is managed by the Secondary Use Service. The ONS mortality data contain information related to a person's death taken from the death certificate for all deaths registered in England and Wales. It contains information such as cause of death, date, and place of death for neonatal and non-neonatal deaths.^12^ It is anticipated that the FIND-AF cohort could provide a setting for cohort RCTs of interventions to prevent AF or detect it early, with aligned mechanistic substudies.

#### Sample size and power calculation

We have the capacity to recruit 500 participants with a higher FIND-AF risk to attend the phenotyping clinic in the first phase of the FIND-AF longitudinal cohort study. To ensure balance in three key baseline variables [FIND-AF risk (lower, higher), dichotomized age (cut off = 75 years), and sex], we aim to recruit at least 80 participants in each subgroup. This will ensure a meaningful comparison of multi-modal phenotyping variables between variables.

#### Analysis plan

A comprehensive statistical analysis plan will be developed by the research team. To assess the prevalence of cardio–renal–metabolic–pulmonary risk factors and cardiovascular diseases, routine data will be compared with diagnosed prevalence after phenotyping. Statistical tests such as the *χ*² test or Fisher’s exact test (for counts less than 5) will be used, with a *P*-value of <0.05 considered statistically significant. We will adhere to the Strengthening the Reporting of Observational Studies in Epidemiology guidelines in reporting quantitative findings^13^ and the CODE-EHR best-practice framework for the use of structured EHRs in clinical research.^14^ Based on the findings from the first phase of phenotyping, more accurate estimations will be possible to plan sample size requirements for RCTs.

#### Patient and public involvement

The FIND-AF Patient and Public Involvement group has informed the FIND-AF programme from inception. They co-designed the study and co-drafted the consent forms and participant information sheets.

#### Ethics and dissemination

The FIND-AF longitudinal cohort study conforms to the Declaration of Helsinki.^15^ The study has ethical approval (the North West—Greater Manchester South Research Ethics Committee reference 23/NW/0180). Findings will be announced at relevant conferences and published in peer-reviewed journals in line with the funder’s open-access policy.

## Discussion

The FIND-AF longitudinal cohort study is a multi-centre, prospective longitudinal cohort study that plans to characterize individuals at risk of AF and form a unique phenotyped cohort from which research studies including RCTs may be undertaken. Recruitment of the first tranche of the FIND-AF cohort of 1955 is expected to be completed by Autumn 2024, of whom about 500 will also be phenotyped.

We selected a prospective observational and linked cohort design because we wished to collect bespoke baseline information (mitigating data-missing-by-design bias and recall bias) as well as providing an efficient mechanism of collecting follow-up data from EHRs. Also, this approach enables a cohort multiple RCT design, whereby the strengths of randomization and observation are combined to enable a potentially more efficient delivery of study.^15^ The study is located in the NHS where there is a publicly funded healthcare system and data are routinely collected in EHRs and administrative databases for all participants, and which may be deterministically linked using each person’s unique NHS number. Our experience of using routine health records data for outcomes capture in a RCT is that there was no loss to follow-up.^16^

Limitations of our approach include (i) the relatively small sample size (although we aim to expand the cohort); (ii) potential delays to data linkage, acquisition, and accuracy; (iii) the potential for selection bias during recruitment; and (iv) that participants may decline recruitment into a RCT after entry into the observational cohort.

## Conclusions

The FIND-AF longitudinal cohort will be a multi-centre, prospective cohort of participants at risk of AF. It will hold high-quality multi-modal data and serve as a powerful tool for understanding the impact of care quality and comorbidities on AF genesis, uncovering actionable targets to prevent AF, and conducting cohort multiple RCTs.

## Data Availability

The data underlying this article will be shared on reasonable request to the chief investigator (C.P.G.) and/or co-investigator (R.N.).
